# The Role of Circadian Rhythms and Sleep in Anorexia Nervosa

**DOI:** 10.1001/jamanetworkopen.2023.50358

**Published:** 2024-01-04

**Authors:** Hannah Wilcox, Valentina Paz, Richa Saxena, John W. Winkelman, Victoria Garfield, Hassan S. Dashti

**Affiliations:** 1 Department of Anesthesia, Critical Care and Pain Medicine, Massachusetts General Hospital and Harvard Medical School, Boston; 2 Center for Genomic Medicine, Massachusetts General Hospital and Harvard Medical School, Boston; 3 Instituto de Psicología Clínica, Facultad de Psicología, Universidad de la República, Montevideo, Uruguay; 4 MRC Unit for Lifelong Health & Ageing, Institute of Cardiovascular Science, University College London, London, United Kingdom; 5 Division of Sleep Medicine, Harvard Medical School, Boston, Massachusetts; 6 Broad Institute, Cambridge, Massachusetts; 7 Sleep Disorders Clinical Research Program, Massachusetts General Hospital and Harvard Medical School, Boston; 8 Division of Nutrition, Harvard Medical School, Boston, Massachusetts

## Abstract

**Importance:**

Observational studies have associated anorexia nervosa with circadian rhythms and sleep traits. However, the direction of causality and the extent of confounding by psychosocial comorbidities in these associations are unknown.

**Objectives:**

To investigate the association between anorexia nervosa and circadian and sleep traits through mendelian randomization and to test the associations between a polygenic risk score (PRS) for anorexia nervosa and sleep disorders in a clinical biobank.

**Design, Setting, and Participants:**

This genetic association study used bidirectional 2-sample mendelian randomization with summary-level genetic associations between anorexia nervosa (from the Psychiatric Genomics Consortium) and chronotype and sleep traits (primarily from the UK Biobank). The inverse-variance weighted method, in addition to other sensitivity approaches, was used. From the clinical Mass General Brigham (MGB) Biobank (n = 47 082), a PRS for anorexia nervosa was calculated for each patient and associations were tested with prevalent sleep disorders derived from electronic health records. Patients were of European ancestry. All analyses were performed between February and August 2023.

**Exposures:**

Genetic instruments for anorexia nervosa, chronotype, daytime napping, daytime sleepiness, insomnia, and sleep duration.

**Main Outcomes and Measures:**

Chronotype, sleep traits, risk of anorexia nervosa, and sleep disorders derived from a clinical biobank.

**Results:**

The anorexia nervosa genome-wide association study included 16 992 cases (87.7%-97.4% female) and 55 525 controls (49.6%-63.4% female). Genetic liability for anorexia nervosa was associated with a more morning chronotype (β = 0.039; 95% CI, 0.006-0.072), and conversely, genetic liability for morning chronotype was associated with increased risk of anorexia nervosa (β = 0.178; 95% CI, 0.042-0.315). Associations were robust in sensitivity and secondary analyses. Genetic liability for insomnia was associated with increased risk of anorexia nervosa (β = 0.369; 95% CI, 0.073-0.666); however, sensitivity analyses indicated bias due to horizontal pleiotropy. The MGB Biobank analysis included 47 082 participants with a mean (SD) age of 60.4 (17.0) years and 25 318 (53.8%) were female. A PRS for anorexia nervosa was associated with organic or persistent insomnia in the MGB Biobank (odds ratio, 1.10; 95% CI, 1.03-1.17). No associations were evident for anorexia nervosa with other sleep traits.

**Conclusions and Relevance:**

The results of this study suggest that in contrast to other metabo-psychiatric diseases, anorexia nervosa is a morningness eating disorder and further corroborate findings implicating insomnia in anorexia nervosa. Future studies in diverse populations and with subtypes of anorexia nervosa are warranted.

## Key Points

**Question:**

What is the association between anorexia nervosa, circadian rhythms, and sleep traits?

**Findings:**

In this genetic association study, a bidirectional association between anorexia nervosa and morning chronotype was found using mendelian randomization. In addition, an association between anorexia nervosa and insomnia was found using mendelian randomization and a polygenic risk score for anorexia nervosa in a clinical biobank.

**Meaning:**

These findings suggest that anorexia nervosa is a morningness eating disorder, in contrast to most other evening-based psychiatric diseases, and corroborate the association between anorexia nervosa and insomnia, concordant with earlier observational studies.

## Teaser

This genetic association study investigates the association between anorexia nervosa and circadian and sleep traits through mendelian randomization and tests the associations between a polygenic risk score for anorexia nervosa and sleep disorders in a clinical biobank.

## Introduction

The circadian clock is the intrinsic biological system that produces 24-hour cyclical processes called circadian rhythms.^1,2^ Circadian rhythms affect a wide spectrum of human physiology, including cycles in body temperature and sleep-wake patterns. Chronotype, the preference for morningness or eveningness, is a correlate of circadian rhythms and an individual’s propensity for earlier or later timing for sleep and other activities and is associated with psychological, neurological, gastrointestinal, and respiratory disorders.^3,4^

There is a bidirectional association between circadian rhythms and psychiatric disorders. For example, tendency for an evening chronotype is associated with depression and schizophrenia.^5,6^ Conversely, bipolar disorder, anxiety, and schizophrenia onset result in alterations in the rhythmicity of core clock genes, and interventions that target the circadian system, such as bright light therapy, are considered valuable treatments.^7,8,9^ There is also some evidence implicating the circadian system in eating disorders, primarily centered on the link between binge eating disorder and evening chronotype in alignment with other psychiatric disorders.^10,11,12^ Characteristics of generally healthy adults with an evening chronotype, such as emotional eating, later food timing, and impulsivity, further support the role of eveningness tendency in binge eating–like behaviors.^13,14^ However, the relevance of the circadian system in anorexia nervosa remains largely unknown.

Anorexia nervosa is a major eating disorder characterized by severe food intake restriction, weight loss, and intense fear of weight gain^15^ and has among the highest mortality rates of all psychiatric diseases.^16^ Some evidence suggests the role of the circadian clock: for example, morningness tendency, with lack of appetite, of which the most prominent is the well-recognized morning anorexia and evening hyperphagia dichotomy that characterizes patients with night eating syndrome,^17^ but also with evening preference in small observational studies.^18^ Additional evidence includes early morning awakening insomnia among people with anorexia nervosa, indicative of perturbations in sleep-wake cycles.^19^ Directionality of the association and whether any effects are causal or mediated by mood and anxiety pathophysiology that often cluster with anorexia nervosa remain unknown.^20^

Genetic analyses can further interrogate the link between anorexia nervosa and circadian and sleep traits. Genome-wide association studies (GWAS) for anorexia nervosa revealed several loci.^21,22^ Mendelian randomization (MR) uses genetic variants that are robustly associated with a trait to examine potential causal effects on outcomes of interest.^23^ Due to the properties of genetic variants, MR is less likely to be affected by unmeasured confounding factors or reverse causation than observational studies. The application of MR has provided additional evidence supporting observational data on hypoleptinemia among patients with anorexia nervosa.^24,25^ It is also speculated that there is a bidirectional association between sleep and eating processes, such that eating pathology disrupts sleep, and conversely, dysregulation in sleep influences eating behaviors.^26^ The aim of this study was to investigate the bidirectional association between anorexia nervosa and chronotype and other sleep traits through MR and a polygenic risk score (PRS) for anorexia nervosa.

## Methods

### GWAS for Anorexia Nervosa

This study followed the Strengthening the Reporting of Observational Studies in Epidemiology Using Mendelian Randomization (STROBE-MR) reporting guidelines.^31^ The largest GWAS meta-analysis for anorexia nervosa from the Eating Disorders Working Group of the Psychiatric Genomics Consortium aggregated data from participants of European ancestry across 33 data sets and included 16 992 cases and 55 525 controls, of which 87.7% to 97.4% of cases were female, 49.6% to 63.4% of controls were female, and 5.3% of the samples were from the UK Biobank.^22^ The case definition of anorexia nervosa was based on a lifetime diagnosis of anorexia nervosa from hospital or register records, structured clinical interviews, online questionnaires based on standardized criteria (ie, *Diagnostic and Statistical Manual of Mental Disorders* [Third Edition Revised]; *Diagnostic and Statistical Manual of Mental Disorders* [Fourth Edition]; *International Classification of Diseases, Eighth Revision*;* International Classification of Diseases, Ninth Revision *[*ICD-9*]; or *International Statistical Classification of Diseases and Related Health Problems, Tenth Revision *[*ICD-10*]), or a self-reported diagnosis. The GWAS identified 8 independent genome-wide significant (*P* < 5 × 10^−8^) single-nucleotide variants (SNVs). SNV-based heritability was estimated at 11% to 17%, reflecting the polygenicity of anorexia nervosa.

### GWAS for Self-Reported Chronotype and Sleep Traits

The primary outcome was chronotype. Summary statistics for the largest GWAS of European ancestry predominantly from the UK Biobank for chronotype (morning preference) was retrieved from the Sleep Disorder Knowledge Portal^27^ (eMethods in Supplement 1). Four sleep traits were also considered secondary outcomes, including daytime napping, daytime sleepiness, and sleep duration from the Sleep Disorder Knowledge Portal^27^ and insomnia from the Center for Neurogenomics and Cognitive Research in Amsterdam.^28^ Traits were determined using primarily self-reported data (eMethods in Supplement 1).

### Statistical Analysis

#### MR Primary Analyses

Bidirectional 2-sample MR between anorexia nervosa and chronotype and sleep traits was conducted using the largest publicly accessible GWAS studies in participants of European ancestry (eTable 1 in Supplement 2) using the “TwoSampleMR” R package. All data sources obtained participant informed consent and relevant ethical approval; as we used publicly available data which does not constitute human participant research, per the US code of federal regulations (45 CFR 46.102), no additional institutional review board approvals were necessary. The primary analysis considered chronotype (morning preference) and 4 secondary sleep traits: daytime napping, daytime sleepiness, insomnia, and sleep duration. For each trait, a genetic instrument was generated using lead GWAS variants (eTables 2-15 in Supplement 2), and standard data harmonization was conducted (eMethods in Supplement 1). The inverse variance weighted (IVW) regression method was used as the primary analysis. The IVW method yields an unbiased estimate in the absence of horizontal pleiotropy.^23,29^ A range of sensitivity analyses robust to the presence of horizontal pleiotropy were tested, including MR-Egger and weighted median estimator (WME) methods. Consistency in effect sizes and overlap of 95% CIs were examined.^30^ A threshold of .05 was used for statistical significance in the MR for our primary analysis with chronotype and secondary analyses with sleep traits. All analyses were performed between February and August 2023.

#### MR Sensitivity and Secondary Analyses

For any significant finding from the IVW analyses, additional sensitivity analyses were conducted including MR pleiotropy residual sum and outlier (PRESSO),^32^ leave-one-out analysis,^29^ MR-Egger intercept,^29^ *I*^2^ quantification,^33^ and a Steiger test^34^ (eMethods in Supplement 1). Secondary analyses were conducted to examine the robustness of the findings including MR analyses for a binary self-report chronotype variable and 3 objective measures of chronotype,^35^ and MR using female-only summary statistics from the insomnia GWAS.^28^

#### PRS for Anorexia Nervosa in the Mass General Brigham Biobank

The Mass General Brigham (MGB) Biobank is the health care enterprise clinical biobank from the MGB health care network in Boston, Massachusetts, and links electronic health records to genetics and lifestyle data. The present protocol was approved by the MGB institutional review board. Patients provided blood samples for genotyping with written consent (eMethods in Supplement 1). All genetic analyses were restricted to participants of European ancestry. A PRS for anorexia nervosa based on effect estimates from the recent meta-analysis GWAS for anorexia nervosa of European ancestry was calculated for each patient.^22^ The PRS was generated using PRS–continuous shrinkage (PRS-CS) based on bayesian regression that places a continuous shrinkage prior on SNV effect sizes.^36^ The UKBiobank EUR-ancestry linkage disequilibrium (LD) panel was set. A total of 1 734 712 SNVs were retained in the PRS following clumping. The PRS was standardized with a mean of 0 and an SD of 1.

Disease designation for anorexia nervosa and sleep disorders was based on PheCode classifications, which aggregate clinically relevant *ICD-9* and *ICD-10* billing codes into disease groups as described in the PheCode catalog^37^ (eMethods in Supplement 1). Anorexia nervosa cases were based on the anorexia nervosa-specific code (305.21), which uses *ICD-9* billing code 307.1 and *ICD-10* billing code F50.0. Association analyses for the anorexia nervosa PRS were conducted for anorexia nervosa and sleep disorders with at least 1% prevalent cases, using the PheWAS R package.^38^ Associations were tested using logistic regression models with adjustments for age, sex, genotyping array, batch, and 10 principal components of ancestry to account for genetic substructure (minimal model), then further adjusted for body mass index, employment status, smoking status, exercise, alcohol intake, and education attainment (fully adjusted model). Significance was considered at the Bonferroni threshold for 9 tested sleep disease outcomes (*P* < .05 / 9 tests).

Enrolled patients were invited to complete an optional Health Information Questionnaire, which included questions on sleep habits (eMethods in Supplement 1). The following circadian and sleep variables from self-report were examined for associations with the PRS: time in bed, sleep debt, sleep midpoint, and social jetlag. Associations were tested using adjusted linear regression models as described previously, and in sensitivity analyses, further adjusted for PheCodes for depression (296.2) and anxiety (300.1), 2 prevalent comorbidities.^39^ Significance was considered at the Bonferroni threshold for 4 circadian and sleep outcome traits (*P* < .05 / 4 tests). All analyses were conducted using R software version 4.2.3 (R Project for Statistical Computing).

## Results

### MR Analyses for Anorexia Nervosa and Chronotype

**Figure 1.**
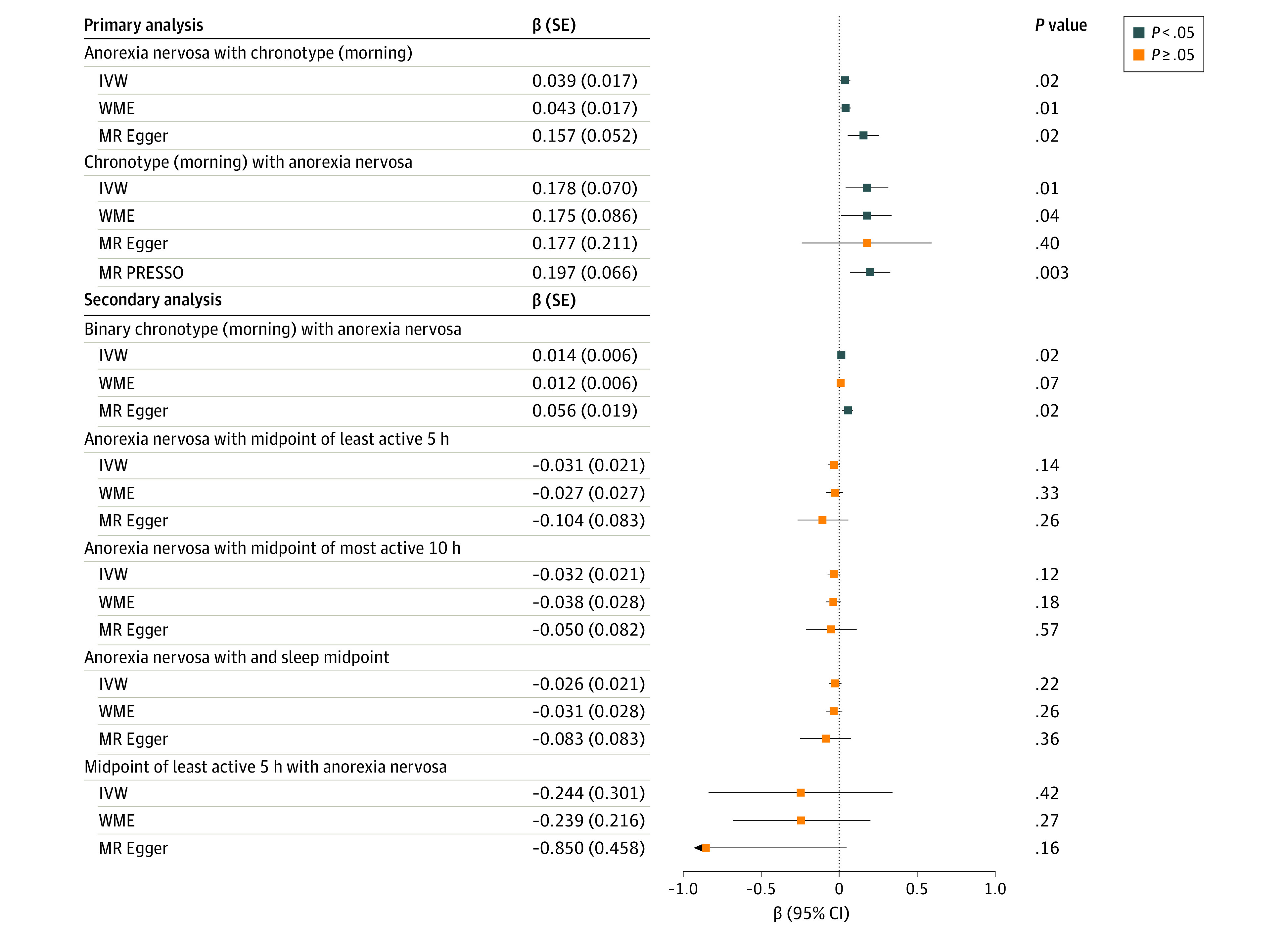
Forest Plot of Mendelian Randomization (MR) Primary, Sensitivity, and Secondary Results for the Associations Between Anorexia Nervosa and Morning Chronotype Forest plot of MR primary, sensitivity, and secondary results for the associations between anorexia nervosa and chronotype (morning), including accelerometer-derived objective chronotype measures of midpoint of least active 5 hours, midpoint of most active 10 hours, and sleep midpoint. β Values reflect the difference in the outcome for each 1-unit increase in the exposure, with blue boxes denoting statistically significant results. Error bars represent 95% CIs. Primary analyses included inverse variance weighted (IVW) MR, weighted median estimator (WME), and MR-Egger, and sensitivity analyses included MR pleiotropy residual sum and outlier (PRESSO).

The anorexia nervosa GWAS included 16 992 cases (87.7%-97.4% female) and 55 525 controls (49.6%-63.4% female). We first conducted bidirectional 2-sample MR between anorexia nervosa and chronotype. Using IVW MR, we found a bidirectional association between anorexia nervosa and morning chronotype (Table 1 and Figure 1). Genetic liability for anorexia nervosa was associated with a more morning chronotype (β = 0.039; 95% CI, 0.006-0.072), and conversely, genetic liability for morning chronotype was associated with increased risk of anorexia nervosa (β = 0.178; 95% CI, 0.042-0.315). Bidirectional effect sizes were consistent using MR-Egger and WME (Table 1 and Figure 1).

**Table 1.** MR Primary Analyses Results for the Associations Between Anorexia Nervosa and Chronotype and Sleep Traits

Exposure	SNVs, No.	IVW		WME		MR-Egger	
		β (SE)^a^	*P* value	β (SE)^a^	*P* value	β (SE)^a^	*P* value
**Anorexia nervosa with circadian and sleep traits**							
Chronotype (morning)	8	0.039 (0.017)	.02	0.043 (0.017)	.01	0.157 (0.052)	.02
Daytime napping	8	−0.003 (0.015)	.83	0.011 (0.009)	.23	0.040 (0.061)	.54
Daytime sleepiness	8	−0.008 (0.006)	.16	−0.005 (0.007)	.41	0.014 (0.023)	.57
Insomnia	8	−0.005 (0.037)	.89	−0.004 (0.035)	.91	0.311 (0.088)	.01
Sleep duration	8	−0.020 (0.011)	.08	−0.021 (0.014)	.13	−0.094 (0.039)	.05
**Circadian and sleep traits with anorexia nervosa**							
Chronotype (morning)	315	0.178 (0.070)	.01	0.175 (0.086)	.04	0.177 (0.211)	.40
Daytime napping	112	0.351 (0.244)	.15	0.117 (0.262)	.66	0.433 (0.869)	.62
Daytime sleepiness	40	0.225 (0.469)	.63	0.752 (0.518)	.15	1.422 (1.767)	.43
Insomnia	467	0.339 (0.147)	.02	0.306 (0.172)	.08	−0.490 (0.568)	.39
Sleep duration	73	−0.084 (0.170)	.62	−0.164 (0.187)	.38	−0.681 (0.642)	.29

Abbreviations: IVW, inverse variance weighted; MR, mendelian randomization; SNV, single-nucleotide variant; WME, weighted median estimator.

^a^ β Values reflect the difference in the outcome for each 1-unit increase in the exposure.

We performed a series of sensitivity analyses to further assess the presence of horizontal pleiotropy. Results from the MR-Egger intercept were not significant for horizontal pleiotropy with anorexia nervosa as the exposure or with chronotype as the exposure. Findings from MR PRESSO suggested possible pleiotropy with chronotype as the exposure (*P* < .001), however this was mitigated by removing 3 outlier variants, resulting in estimates consistent with the main analysis (β = 0.197; 95% CI, 0.067-0.327) (Figure 1; eTable 16 in Supplement 2). Assessment of horizontal pleiotropy using MR PRESSO was not significant for pleiotropy with anorexia nervosa as the exposure. Leave-one-out analysis did not suggest undue influence by single outliers or systemic bias (eTables 17-18 in Supplement 2). The calculated *I*^2^ quantity was 49.6% with anorexia nervosa as the exposure and 48.2% with chronotype as the exposure. The Steiger test held true in both directions.

The observed direction of the association between chronotype and anorexia nervosa was consistent in secondary analyses using summary statistics for binary chronotype (morning vs evening preference) as the outcome (β = 0.014; 95% CI, 0.002-0.026) (Figure 1 and Table 2). In sensitivity analyses, there was no evidence of horizontal pleiotropy using the MR-Egger intercept test or MR PRESSO. Leave-one-out analysis did not suggest undue influence by single outliers or systemic bias (eTable 19 in Supplement 2). The *I*^2^ quantity was calculated as 41.0%, and the Steiger test held true.

**Table 2.** MR Secondary Analyses Results for the Associations Between Anorexia Nervosa and Chronotype and Sleep Traits

Exposure	SNVs, No.	IVW		WME		MR-Egger	
		β (SE)^a^	*P* value	β (SE)^a^	*P* value	β (SE)^a^	*P* value
Anorexia nervosa with chronotype							
Binary chronotype (morning)	8	0.014 (0.006)	.02	0.012 (0.006)	.07	0.056 (0.019)	.02
Midpoint of least active 5 h, accelerometer	8	−0.031 (0.021)	.14	−0.027 (0.027)	.33	−0.104 (0.083)	.26
Midpoint of most active 10 h, accelerometer	8	−0.032 (0.021)	.12	−0.038 (0.028)	.18	−0.050 (0.082)	.57
Sleep midpoint, accelerometer	8	−0.026 (0.021)	.22	−0.031 (0.028)	.26	−0.083 (0.083)	.36
Chronotype and sleep traits with anorexia nervosa							
Midpoint of least active 5 h, accelerometer	5	−0.244 (0.301)	.42	−0.239 (0.216)	.27	−0.850 (0.458)	.16
Female-only insomnia	272	0.324 (0.157)	.04	0.289 (0.171)	.09	−0.063 (0.740)	.93

Abbreviations: IVW, inverse variance weighted; MR, mendelian randomization; SNV, single-nucleotide variant; WME, weighted median estimator.

^a^ β Values reflect the difference in the outcome for each 1-unit increase in the exposure.

We found no evidence for the association between anorexia nervosa and chronotype using accelerometer-derived sleep timing (Figure 1 and Table 2). However, MR direction of associations for risk of anorexia nervosa were consistent.

### MR Analyses for Anorexia Nervosa and Sleep Traits

**Figure 2.**
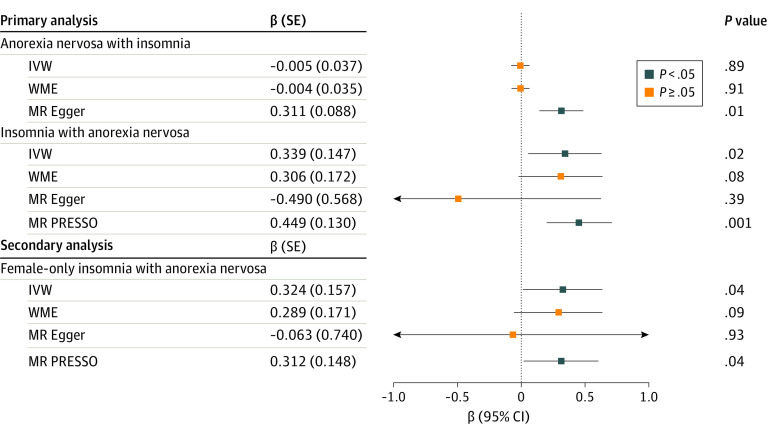
Forest Plot of Mendelian Randomization (MR) Primary, Sensitivity, and Secondary Results for Associations Between Anorexia Nervosa and Insomnia β Values reflect the difference in the outcome for each 1-unit increase in the exposure, with blue boxes denoting statistically significant results. Error bars represent 95% CIs. Primary analyses included inverse variance weighted (IVW) MR, weighted median estimator (WME), and MR-Egger, and sensitivity analyses included MR pleiotropy residual sum and outlier (PRESSO).

In MR, we found an association between insomnia and increased risk of anorexia nervosa, and no associations between anorexia nervosa and daytime napping, daytime sleepiness, or sleep duration (Table 1). Genetic liability for insomnia was associated with increased risk of anorexia nervosa using IVW MR (β = 0.339; 95% CI, 0.052-0.627); however, no association was observed for the genetic liability of anorexia nervosa with increased risk of insomnia (Table 1 and Figure 2). In sensitivity analyses, effect sizes were consistent using WME but not MR-Egger models. Results from the MR-Egger intercept were not significant for horizontal pleiotropy. However, results from MR PRESSO were significant (*P* < .001), and subsequently mitigated through removing 6 outlier variants resulting in estimates consistent with the main analysis (β = 0.449; 95% CI, 0.195-0.703) (Figure 2; eTable 16 in Supplement 2). Leave-one-out analysis did not suggest influence by single outliers or systemic bias (eTable 20 in Supplement 2). The *I*^2^ quantity was calculated as 51.5%, and the Steiger test proved false, suggesting the direction of causality of insomnia on anorexia nervosa cannot be ascertained.

When using effect sizes from the female-only insomnia GWAS, we also found that genetic liability for insomnia was associated with increased risk of anorexia nervosa using IVW MR (β = 0.324; 95% CI, 0.015-0.632) (Table 2 and Figure 2). Results from the MR-Egger intercept were not significant for horizontal pleiotropy. However, results from MR PRESSO were significant (*P* < .001) and subsequently mitigated through removing 4 outlier variants, resulting in estimates consistent with the main analyses (β = 0.312; 95% CI, 0.021-0.603) (Figure 2; eTable 16 in Supplement 2). Leave-one-out analysis did not suggest influence by single outliers or systemic bias (eTable 21 in Supplement 2). The *I*^2^ quantity was calculated as 54.0%, and the Steiger test was also false.

### Anorexia Nervosa in the MGB Biobank

To further examine associations between anorexia nervosa and sleep disorders in a disease-enriched and independent health enterprise clinical biobank, we conducted a series of analyses in the MGB Biobank. A total of 47 082 adult patients of European ancestry with genetic data were included in the MGB Biobank analyses (mean [SD] age, 60.4 [17.0] years; 25 318 [53.8%] female). We first examined associations between the anorexia nervosa PRS and anorexia nervosa diagnosis based on PheCodes. Each SD increase in the PRS was associated with 36% higher odds of anorexia nervosa (odds ratio [OR], 1.36; 95% CI, 1.14-1.63) (Table 3). In association analyses with 9 prevalent sleep disorders, we found associations with higher organic or persistent insomnia. Each additional SD in the anorexia nervosa PRS was associated with 10% higher odds for organic or persistent insomnia (OR, 1.10; 95% CI, 1.03-1.17), which remained unchanged when accounting for lifestyle factors. Associations with other sleep disorders including sleep apnea and restless legs syndrome were not significant. In addition, among patients with self-reported sleep habits (n = 16 109), there were no associations evident between the anorexia nervosa PRS and time in bed, sleep midpoint, sleep debt, or social jetlag (eTable 22 in Supplement 2).

**Table 3.** Associations Between Polygenic Risk Score for Anorexia Nervosa and Anorexia Nervosa and Sleep Disorders in the Mass General Brigham Biobank (n = 47 082)^a^

Disease description	Phecode	Model 1			Model 2		
		No. of cases/No. of controls	OR (95% CI)	*P* value	No. of cases/No. of controls	OR (95% CI)	*P* value
Anorexia nervosa	305.21	182/17 106	1.36 (1.14-1.63)	<.001	57/6417	NA	NA
Organic or persistent insomnia	327.41	1343/31 569	1.10 (1.03-1.17)	.005	403/11 266	1.18 (1.05-1.33)	.006
Insomnia	327.4	5748/35 974	1.04 (1.00-1.07)	.04	1637/11 266	1.01 (0.94-1.07)	.87
Restless legs syndrome	327.71	1281/31 507	1.07 (1.00-1.14)	.06	397/11 266	0.99 (0.88-1.11)	.84
Central/nonobstructive sleep apnea	327.31	555/30 781	0.95 (0.86-1.05)	.32	172/11 266	0.83 (0.70-0.99)	.04
Sleep apnea	327.3	4161/34 387	0.99 (0.95-1.03)	.66	1190/11 266	1.02 (0.95-1.10)	.64
Parasomnia	327.5	497/30 723	1.02 (0.92-1.13)	.70	154/11 266	1.01 (0.83-1.21)	.95
Obstructive sleep apnea	327.32	6722/36 948	1.01 (0.97-1.04)	.71	1952/11 266	1.01 (0.96-1.08)	.63
Hypersomnia	327.1	989/31 215	1.01 (0.94-1.09)	.72	314/11 266	1.03 (0.90-1.18)	.65
Sleep disorders	327	3048/33 274	1.01 (0.96-1.05)	.73	898/11 266	1.00 (0.92-1.08)	.99

Abbreviations: NA, not applicable; OR, odds ratio.

^a^ Effect sizes are per SD increase in the polygenic risk score for anorexia nervosa. Associations were tested using adjusted logistic regression models. Model 1 was adjusted for age, sex, genotyping array, batch, and 10 principal components of ancestry (minimal model). Model 2 was further adjusted for body mass index, employment status, smoking status, exercise, alcohol intake, and education attainment (fully adjusted model). Findings for anorexia nervosa are missing for model 2 due to insufficient number of cases. Significance was considered at the Bonferroni threshold for 9 tested sleep disease outcomes (*P* < .05 / 9 tests).

## Discussion

Using MR, we provide evidence for bidirectional associations between anorexia nervosa and morning chronotype, which were consistent in a series of sensitivity and secondary analyses. We also found associations between insomnia and higher risk for anorexia nervosa concordant with earlier observational studies; however, these effect sizes were possibly confounded by psychosocial comorbidities. We did not find associations between anorexia nervosa and other sleep traits, including daytime napping, daytime sleepiness, and sleep duration.

The bidirectional association between anorexia nervosa and morning chronotype proved robust to sensitivity analyses using alternative approaches to ascertain chronotype and to major bias due to pleiotropy. Results using accelerometer-derived measures of chronotype were consistent with findings from self-report implicating morningness with anorexia nervosa but were ultimately not significant likely due to limited power (85 670 participants in the accelerometer GWAS in comparison with 449 734 participants in the self-report GWAS). In a clinical biobank, we did not observe associations between the anorexia nervosa PRS and self-reported sleep midpoint, a measure of chronotype, possibly due to limited power or confounding comorbidities. As many as 62% and 54% of people diagnosed with an eating disorder also have an anxiety disorder and a mood disorder such as depression, respectively.^39^ Anxiety and depression, and most other psychiatric diseases, have consistently been associated with eveningness, potentially confounding previous findings using observational data.^40,41^ Furthermore, anorexia nervosa is most common at a younger age, peaking in onset around ages 15 to 16 years, when people experience a delay in chronotype toward eveningness.^42,43^ MR mitigates these confounding effects through the use of random genetic variation, allowing for robust analysis of the connection between anorexia nervosa and chronotype independent of psychosocial comorbidities and resolution of seemingly conflicting evidence from observational studies.

Our findings suggest that anorexia nervosa is another eating disorder with likely a circadian feature. First, in observational data, anorexia nervosa was associated with breakfast and lunch skipping, reinforcing the connection between anorexia and food temporality.^44^ Second, morning bright light therapy was deemed less effective in anorexia nervosa treatment compared with binge eating disorders and bulimia nervosa, 2 eating disorders presumed to be evening based.^7,9^ Third, compared with healthy controls, malnourished patients with anorexia nervosa have an enhanced and advanced cortisol awakening response often exhibited with earlier chronotype.^45,46^ Lastly, patients with anorexia nervosa often experience early morning awakening insomnia indicative of perturbations in sleep-wake cycles.^19^ Contradictory evidence from observational data linking anorexia nervosa with evening chronotype^18^ or showing no associations with sleep timing^47^ are small and may be confounded by psychosocial comorbidities. The precise role of the circadian system in the etiology of anorexia nervosa remains unclear.

In addition, we found a potential association between insomnia and increased risk of anorexia nervosa. Although findings from the IVW method were inconsistent in sensitivity analyses using MR-Egger, a low *I*^2^ test statistic^48^ indicated that the MR-Egger result was likely biased.^49^ Our findings provide evidence for a robust association between insomnia and anorexia nervosa without bias due to horizontal pleiotropy; however, the direction of causality cannot be proven. The results from MR were further corroborated in a clinical biobank using a PRS for anorexia nervosa, in which we observed positive associations between anorexia nervosa and organic or persistent insomnia. Directionality cannot be inferred from this cross-sectional analysis.

The clinical implications of our novel bidirectional chronotype findings are unclear; however, our study provides several opportunities for future research into the prevention and treatment of anorexia nervosa. Our findings suggest the role of morningness as an underrecognized risk factor for anorexia nervosa whose importance should be further investigated in the context of other established risk factors. If deemed a relevant risk factor, interventions promoting later sleep schedules may be considered to ameliorate risk conferred by a morning chronotype. Our findings also direct research into circadian-based mechanisms for future anorexia nervosa treatment. Unlike morning bright light therapy, which has resulted in mixed findings in preliminary studies, bright light therapy in the evening, as is recommended for patients with early morning awakening insomnia, may be efficacious in the prevention and treatment of anorexia nervosa.^50^ Whether this would impact transdiagnostic treatment of more evening-based psychiatric comorbidities, eg, depression, remains unknown. Replication analyses and mechanistic studies are crucial to warrant any circadian-based therapies suggested by our findings.

This study has several strengths. We leveraged the largest GWAS for anorexia nervosa, chronotype, and sleep traits available at the time of analysis. In addition, we provided evidence of robust replication of the anorexia nervosa genetic variants in an independent clinical biobank facilitated by a higher prevalence of anorexia nervosa (0.3% of samples) in our clinical MGB Biobank, far exceeding other biobanks in previous studies including the Bio*Me* Biobank (0.02% of samples).^51^ However, this prevalence is likely not sufficient to interrogate associations between anorexia nervosa diagnosis and other genetic variants, such as a PRS for chronotype or insomnia.

### Limitations

There are some important limitations to note. As the anorexia nervosa GWAS focused on broadly defined anorexia nervosa based on billing codes and/or self-report, we were unable to interrogate anorexia nervosa subtypes, such as restrictive, binge-eating/purging, or atypical anorexia nervosa.^22^ Our analyses only considered participants of European ancestry, as the genetic discoveries were from predominantly European cohorts and the transferability of variants into non-European populations remains unknown.^52^ It is worth noting that anorexia nervosa prevalence remains currently highest among European and Western cultures, is rapidly rising among young adolescent girls,^53^ and is likely underdiagnosed in men and individuals from minoritized racial and ethnic groups,^52^ necessitating future large investigations on early or late onset anorexia nervosa^54^ and anorexia in non-European populations. We presented MR *P* values unadjusted for multiple testing because our study is exploratory in nature. Therefore, we acknowledge that individual findings should be interpreted with caution, and that additional studies are necessary to verify these findings. Additionally, as the anorexia nervosa GWAS included 5.3% samples from the UK Biobank, there was some sample overlap in the 2-sample MR analysis, which may have led to the overestimation of effect sizes.^55^

## Conclusions

Our study found bidirectional associations between anorexia nervosa and morning chronotype, suggesting that in contrast to other metabo-psychiatric diseases, anorexia nervosa is distinctly a morningness eating disorder. In addition, the associations between anorexia nervosa and insomnia corroborate earlier findings.

## Notes

Supplement 1.
eMethods.

Supplement 2. **eTable 1.** Genome-wide association studies for anorexia nervosa and circadian and sleep traits **eTable 2.** SNVs and proxies included in genetic instrument for anorexia nervosa with chronotype **eTable 3.** SNVs and proxies included in genetic instrument for anorexia nervosa with binary chronotype **eTable 4.** SNVs and proxies included in genetic instrument for anorexia nervosa with daytime napping **eTable 5.** SNVs and proxies included in genetic instrument for anorexia nervosa with daytime sleepiness **eTable 6.** SNVs and proxies included in genetic instrument for anorexia nervosa with insomnia **eTable 7.** SNVs and proxies included in genetic instrument for anorexia nervosa with sleep duration **eTable 8.** SNVs and proxies included in genetic instrument for anorexia nervosa with accelerometer derived midpoint of least active 5 hours, midpoint of most active 10 hours, and sleep midpoint **eTable 9.** SNVs and proxies included in genetic instrument for chronotype with anorexia nervosa **eTable 10.** SNVs and proxies included in genetic instrument for daytime napping with anorexia nervosa **eTable 11.** SNVs and proxies included in genetic instrument for daytime sleepiness with anorexia nervosa **eTable 12.** SNVs and proxies included in genetic instrument for insomnia with anorexia nervosa **eTable 13.** SNVs and proxies included in genetic instrument for female only insomnia with anorexia nervosa **eTable 14.** SNVs and proxies included in genetic instrument for sleep duration with anorexia nervosa **eTable 15.** SNVs and proxies included in genetic instrument for accelerometer derived midpoint of least active 5 hours with anorexia nervosa **eTable 16.** Variants identified as outliers by MR-PRESSO **eTable 17.** Leave-one-out analysis for results of anorexia nervosa with chronotype. IVW MR conducted with listed SNV removed in each respective analysis **eTable 18.** Leave-one-out analysis for results of chronotype with anorexia nervosa. IVW MR conducted with listed SNV removed in each respective analysis **eTable 19.** Leave-one-out analysis for results of anorexia nervosa with binary chronotype. IVW MR conducted with listed SNV removed in each respective analysis **eTable 20.** Leave-one-out analysis for results of insomnia with anorexia nervosa. IVW MR conducted with listed SNV removed in each respective analysis **eTable 21.** Leave-one-out analysis for results of female only insomnia with anorexia nervosa. IVW MR conducted with listed SNV removed in each respective analysis **eTable 22.** Association between polygenic risk score for anorexia nervosa and self-reported sleep traits in the Mass General Brigham Biobank (n =16,109)

Supplement 3.
Data Sharing Statement
