# Supplementary material for: The Role of Circadian Rhythms and Sleep in Anorexia Nervosa
Source: JAMA Netw Open. 2024 Jan 4;7(1):e2350358. doi: 10.1001/jamanetworkopen.2023.50358 (PMC10767597; doi:10.1001/jamanetworkopen.2023.50358)
Supplement: Supplement 1. — eMethods. [file jamanetwopen-e2350358-s001.pdf]

Supplemental Online Content

Wilcox H, Paz V, Saxena R, Winkelman JW, Garfield V, Dashti HS. The role of circadian rhythms and sleep in anorexia nervosa. *JAMA Netw Open.* 2024;7(1):e2350358. doi:10.1001/jamanetworkopen.2023.50358

eMethods.

This supplemental material has been provided by the authors to give readers additional information about their work.

© 2024 Wilcox H et al. *JAMA Network Open.*

eMethods.

Genome-wide association studies for self-reported chronotype and sleep traits
Summary statistics for the largest GWAS of European ancestry predominantly from the UK Biobank for chronotype was retrieved from the Sleep Disorder Knowledge Portal[1]. Briefly, the GWAS for *chronotype* included 449,734 participants from the UK Biobank and 248,100 participants from 23andMe[2]. Participants in the UK Biobank responded to the question, "Do you consider yourself to be?" with response options "Definitely a 'morning' person", "More a 'morning' than 'evening' person", "More an 'evening' than a 'morning' person", "Definitely an 'evening' person", "Do not know" or "Prefer not to answer". Participants with neutral responses were excluded. Participants from 23andMe responded to the question, "Are you naturally a night person or a morning person?" twice with first response options "Night owl", "Early bird", and "Neither" and second response options "Night person", "Morning person", "Neither", "It depends" and "I'm not sure". Participants with discordant or neutral responses to both questions were excluded. GWAS was conducted for morning preference as a continuous variable and identified 351 independent genome-wide significant SNPs. In analyses, summary statistics from the UK Biobank only subset were used due to limited public availability of full meta-analysis data. This subset included 449,734 participants with a mean age of 56.8 and 45.7% male.

Four sleep traits were also considered including daytime napping, daytime sleepiness, sleep duration, from the Sleep Disorder Knowledge Portal[1], and insomnia from the Center for Neurogenomics and Cognitive Research in Amsterdam[3]. The GWAS for *daytime napping* included 452,633 participants from the UK Biobank, aged 40-69 with 54% female[4]. Participants were asked "Do you have a nap during the day?", with responses "Never/rarely", "Sometimes", "Usually", or "Prefer not to answer". Responses were treated as a continuous variable and 123 independent genome-wide significant SNPs were identified in GWAS. The GWAS for *daytime sleepiness* included 452,071 participants from the UK Biobank with a mean age of 57.3 and 54% female[5]. Participants responded to the question "How likely are you to doze off or fall asleep during the daytime when you don't mean to? (e.g.: when working, reading or driving)" with responses "never", "sometimes", "often", or "all of the time". Responses were treated as a continuous variable and the GWAS identified 42 genome-wide significant SNPs. The GWAS for *sleep duration* GWAS included 446,118 participants from the UK Biobank with a mean age of 57.3 and 54% female[6]. Participants were asked, "About how many hours sleep do you get in every 24 h? (please include naps)", with responses in hour increments. GWAS identified 78 independent genome-wide significant SNPs. Only effect estimates from the primary GWAS without BMI adjustments were analyzed.

Summary statistics for the largest *insomnia* GWAS were retrieved from the Center for Neurogenomics and Cognitive Research in Amsterdam[3]. The GWAS included 593,724 insomnia cases and 1,771,286 controls from the UK Biobank and 23andMe and identified 554 independent genome-wide significant SNPs. UK Biobank participants were asked the question, "Do you have trouble falling asleep at night or do you wake up in the middle of the night?" with response options "never/rarely", "sometimes", "usually", and "prefer not to answer" with those answering "usually" set as cases and the remaining set as controls. 23andMe participants were asked questions related to seven sleep related traits. Participants with positive response to any of the following questions were considered as cases: 1) 'Have you ever been diagnosed with, or treated for, insomnia?', 2) 'Were you diagnosed with insomnia?', 3) 'Have you ever been diagnosed by a doctor with any of the following neurological conditions?' (Sleep disturbance), 4) 'Do you routinely have trouble getting to sleep at night?', 5) 'What sleep disorders have you been diagnosed with? Please select all that apply.' (Insomnia, trouble falling or staying asleep), 6) 'Have you ever taken these medications?' (Prescription sleep aids) and 7) 'In the last 2 years, have you taken any of these medications?' (Prescription sleep aids). Participants who did not provide either a positive or uncertain answer ('I don't know' or 'I am not sure') in any of the 7 questions nor the followings were considered as controls: 1) 'Have you ever been diagnosed with, or treated for, any of the following conditions?' (Insomnia; Narcolepsy; Sleep apnea; Restless leg syndrome), 2) 'In the past 12 months, have you been newly diagnosed with any of the following conditions by a medical professional?' (Insomnia; Sleep apnea; Migraines), 3) 'Have you ever been diagnosed with or treated for any of the following conditions?' (Posttraumatic stress disorder; Autism; Asperger's; Sleep disorder), 4) 'Have you ever been diagnosed with or treated for a sleep disorder?', and 5) 'Have you ever been diagnosed with or treated for any of the following conditions?' (A sleep disorder). For the analysis of anorexia nervosa on insomnia, summary statistics from the UK Biobank only subset were used due to limited public availability of full meta-analysis data, including 109,548 cases (61% female) and 277,440 controls (51% female).

© 2024 Wilcox H et al. *JAMA Network Open.*

Mendelian randomization primary analyses

Bidirectional two-Sample MR between anorexia nervosa and chronotype and sleep traits was conducted using the largest publicly accessible GWAS studies in participants of European ancestry (as listed in **eTable 1**) using the "TwoSampleMR" R package. The primary analysis considered chronotype and four sleep traits: daytime napping, daytime sleepiness, insomnia, and sleep duration.

Three necessary assumptions for valid causal inference in MR exist: 1) the genetic variants must strongly associate with the trait of interest; 2) the associations between the genetic variants and the outcome are independent of unmeasured confounders; and, 3) the genetic variants affect the outcome only through their effect on the exposure[7]. Only the first assumption can be directly accounted for through use of independent genome-wide significant ($P < 5 \times 10^{-8}$) SNPs as the instrumental variable for exposure. The second and third assumptions are accounted for by tests for horizontal pleiotropy, which occurs if the effects of a genetic variant on an outcome are independent of the exposure, but rather via a secondary risk factor.

For each trait, a genetic instrument was generated using lead GWAS variants (**eTables 2–15**). Wherever necessary (e.g., daytime sleepiness), clumping was performed using an r^2 threshold of 0.1 and kb threshold of 1000. For daytime sleepiness, one variant was removed due to LD with other variants or absence from the LD reference panel. An F-statistic[8] was calculated for the genetic instruments to ensure adequate power of the instrumental variables using the formula $F = (beta/se)^2$ (**eTable 1**). For each variant, exposure and outcome effects were harmonized to the same effect allele. When variants were missing in the outcome dataset, the nearest available proxy SNP in linkage disequilibrium at $r^2 > 0.80$ identified by the NIH LDproxy Tool was used, if available[9] (**eTables 2-15**). Insertion/deletions, variants absent from the reference panel, and palindromic variants (i.e., SNPs whose alleles correspond to nucleotides that pair in forward and reverse coding, such as A/T or C/G alleles) with intermediate allele frequencies (i.e., ~0.50) with ambiguities that cannot be reconciled were excluded. To retain as many genetic variants for anorexia nervosa as possible, palindromic variants with intermediate allele frequencies were retained and manually harmonized in MR analyses with anorexia nervosa as the exposure but were included in MR analyses with anorexia nervosa as the outcome.

Following data harmonization, the inverse variance weighted (IVW) regression method was used as the primary analysis. The IVW method yields an unbiased estimate in the absence of horizontal pleiotropy[7,10]. A range of sensitivity analyses robust to the presence of horizontal pleiotropy were tested, including MR-Egger and weighted median estimator (WME) methods. MR-Egger allows all variants to have pleiotropic effects on the outcome, with a loss in power of the statistical method, and WME allows half of the genetic variants to have pleiotropic effects without a loss of consistency in the MR results. In the absence of unbalanced horizontal pleiotropy, all tests should yield results consistent with the IVW method. However, these approaches are less powerful than the IVW method, and thus consistency in effects and overlap of 95% confidence intervals examined[11]. A threshold of 0.05 was used for statistical significance in MR.

Mendelian randomization sensitivity analyses

For any significant finding from the primary analyses, additional sensitivity analyses were conducted. MR Pleiotropy Residual Sum and Outlier (PRESSO) applies a global distortion test to implement an outlier detection strategy to select and remove potentially pleiotropic variants, providing a method more sensitive to horizontal pleiotropy than MR-Egger[12]. A leave-one-out analysis was also conducted to assess if the results were influenced by singular outliers[10]. Horizontal pleiotropy was further assessed by calculating the MR-Egger intercept, which indicates directional pleiotropy where a nonzero intercept indicates the presence of pleiotropy in the effect of the genetic variant on the exposure[10]. The I^2 quantity was calculated by the formula $I^2 = 100\% \times (Q - df)/Q$, where Q is Cochran's heterogeneity statistic and degrees of freedom (df) to assess the level of heterogeneity[13]. Lastly, a Steiger test was used to examine proper direction of causality in the overall analysis[14].

Mendelian randomization secondary analyses

A series of secondary analyses were conducted to examine the robustness of the findings from the primary MR analyses using complementary datasets for the exposure or outcome. Specifically, MR analyses for a binary self-report chronotype variable and 3 objective measures of chronotype were conducted to further examine the bidirectional associations between anorexia nervosa and chronotype. In the UK Biobank, a binary chronotype variable (e.g., morning vs. evening) was derived where responses of "Definitely a 'morning' person" and "More a 'morning' than 'evening' person" were set to cases and "Definitely an 'evening' person" and "More an 'evening'

than a ‘morning’ person” were set to controls[2]. The sensitivity GWAS included 252,287 cases of morning persons and 150,908 controls. In addition, among a subset of 85,670 participants from the UK Biobank who provided data from a wrist-worn accelerometer[15], objective measures of chronotype (sleep timing) were generated and analyzed in GWAS. Measures of chronotype included *midpoint of the least active 5 hours (L5)* reflecting the timing of the least activity during a 24-hour period, *midpoint of the most active 10 hours (M10)* reflecting the timing of the most activity during a 24-hour period, and *sleep midpoint* reflecting the midpoint of the time between sleep onset and offset, giving objective circadian measures[2]. Unidirectional MR were conducted for anorexia nervosa on the *midpoint of the most active 10 hours* and *sleep midpoint* considering the limited number of variants identified in GWAS which precludes reverse MR analyses[15].

To further examine the findings between anorexia nervosa and insomnia in secondary analyses, female-only MR was conducted using the female-only summary statistics from the insomnia GWAS[3]. As the female specific anorexia nervosa GWAS is not publicly available and the anorexia nervosa cases were approximately 97.1% female, the sex-combined summary statistics were used for anorexia nervosa. For each analysis, the same MR methods described previously were conducted.

Polygenic risk score for anorexia nervosa in the Mass General Brigham Biobank

The Mass General Brigham (MGB) Biobank is the healthcare enterprise clinical biobank from the MGB healthcare network in Boston, Massachusetts. The biobank links electronic health records (EHR) to genetics and lifestyle data aggregated from over 140,000 patients since 2009. As described previously, patients were recruited in-person from multiple MGB community-based primary care facilities and specialty tertiary care centers as well as through the patient portal[16]. Written consent was obtained from all patients upon enrollment. At the time of analysis (early 2023), 140,915 patients had consented.

DNA was genotyped using the Infinium Global Screening Array-24 version 2.0 (Illumina, San Diego, SD). Briefly, imputation was performed using the Michigan Imputation server with the Trans-Omics for Precision Medicine (TOPMed) (version r2) reference panel, and haplotype phasing was performed using Eagle version 2.3[17,18]. Low-quality genetic markers and samples were excluded as previously described. One sample from each pair of related participants (kinship > 0.0625) was retained for analysis. Principal components of ancestry were computed using TRACE and the Human Genome Diversity Project to correct for the population substructure[19,20].

Disease designation for anorexia nervosa and sleep disorders was based on PheCode classifications which aggregates clinically relevant International Classification of Diseases (ICD)-9/-10 billing codes into disease groups as described in the PheCode catalog[21]. For any PheCode, patients with at least two codes on two separate dates were considered cases, whereas those with no relevant codes were presumed to be free of the disease and thus considered controls. Patients with only one relevant code or with any code for an exclusionary disease (e.g., a related condition that may contaminate the grouping of cases and controls as determined in the PheWAS catalog), were excluded from that analysis[21]. Anorexia nervosa cases were based on the anorexia nervosa-specific code (305.21) which uses anorexia nervosa billing codes ICD-9 307.1 and ICD-10 F50.0 and excludes relevant diagnoses including eating disturbance NOS (ICD-9 307.50) feeding problem (ICD-9 783.3) of nonorganic origin (ICD-9 307.59) loss of appetite (ICD-9 783.0) of nonorganic origin (ICD-9 307.59). In aggregate, biobank patients included in the present analysis had a total of 41,485,748 diagnostic codes corresponding to 2,519,179 instances of PheCodes with at least 2 distinct diagnostic codes.

All enrolled patients were invited to complete an optional Health Information Questionnaire, which included questions on sleep habits. Patients were asked “In considering your longest sleep period, what time do you usually go to bed on weekdays or work or school days [also weekends or days off]?” and “In considering your longest sleep period, what time do you usually wake up on weekdays or work or school days [also weekends or days off]?” Responses were in half-hour increments. From responses to the sleep habits questions, we calculated the following: (1) time in bed as the weighted average weekly time in bed with 5/7 weighting for weekdays and 2/7 for weekends; (2) sleep debt (or regularity) as the absolute value of the difference between weekday and weekend time in bed; (3) sleep midpoint as the midpoint of bed and wake times on weekends[22], and; (4) social jetlag as the absolute difference in weekend and weekday sleep midpoint[22]. At the time of analysis, among genotyped participants, a total of 16,109 (34.2%) responded to all 4 sleep habits questions. As previously conducted[22], bedtimes between 08:00 am and 02:00 pm and wake times between 06:00 pm and 12:00 am, likely resulting from am/pm misreporting, and improbable time in bed <3 or >18 hours were set to missing.

© 2024 Wilcox H et al. *JAMA Network Open.*

eReferences.

1. Costanzo MC, von Grotthuss M, Massung J, et al. The Type 2 Diabetes Knowledge Portal: An open access genetic resource dedicated to type 2 diabetes and related traits. *Cell Metab*. 2023;35(4):695-710.e6.

2. Jones SE, Lane JM, Wood AR, et al. Genome-wide association analyses of chronotype in 697,828 individuals provides insights into circadian rhythms. *Nat Commun*. 2019;10(1):343.

3. Watanabe K, Jansen PR, Savage JE, et al. Genome-wide meta-analysis of insomnia prioritizes genes associated with metabolic and psychiatric pathways. *Nat Genet*. 2022;54(8):1125-1132.

4. Dashti HS, Daghlas I, Lane JM, et al. Genetic determinants of daytime napping and effects on cardiometabolic health. *Nat Commun*. 2021;12(1):900.

5. Wang H, Lane JM, Jones SE, et al. Genome-wide association analysis of self-reported daytime sleepiness identifies 42 loci that suggest biological subtypes. *Nat Commun*. 2019;10(1):3503.

6. Dashti HS, Jones SE, Wood AR, et al. Genome-wide association study identifies genetic loci for self-reported habitual sleep duration supported by accelerometer-derived estimates. *Nat Commun*. 2019;10(1):1100.

7. Davies NM, Holmes MV, Davey Smith G. Reading Mendelian randomisation studies: a guide, glossary, and checklist for clinicians. *BMJ*. 2018;362:k601.

8. Garfield V, Salzmann A, Burgess S, Chaturvedi N. A Guide for Selection of Genetic Instruments in Mendelian Randomization Studies of Type 2 Diabetes and HbA1c: Toward an Integrated Approach. *Diabetes*. 2023;72(2):175-183.

9. Machiela MJ, Chanock SJ. LDlink: a web-based application for exploring population-specific haplotype structure and linking correlated alleles of possible functional variants. *Bioinformatics*. 2015;31(21):3555-3557.

10. Burgess S, Thompson SG. Interpreting findings from Mendelian randomization using the MR-Egger method. *Eur J Epidemiol*. 2017;32(5):377-389.

11. Bowden J, Davey Smith G, Burgess S. Mendelian randomization with invalid instruments: effect estimation and bias detection through Egger regression. *Int J Epidemiol*. 2015;44(2):512-525.

12. Verbanck M, Chen CY, Neale B, Do R. Detection of widespread horizontal pleiotropy in causal relationships inferred from Mendelian randomization between complex traits and diseases. *Nat Genet*. 2018;50(5):693-698.

13. Higgins JPT, Thompson SG, Deeks JJ, Altman DG. Measuring inconsistency in meta-analyses. *BMJ*. 2003;327(7414):557-560.

14. Hemani G, Tilling K, Davey Smith G. Orienting the causal relationship between imprecisely measured traits using GWAS summary data. *PLoS Genet*. 2017;13(11):e1007081.

15. Jones SE, van Hees VT, Mazzotti DR, et al. Genetic studies of accelerometer-based sleep measures yield new insights into human sleep behaviour. *Nat Commun*. 2019;10(1):1585.

16. Karlson EW, Boutin NT, Hoffnagle AG, Allen NL. Building the Partners HealthCare Biobank at Partners Personalized Medicine: Informed Consent, Return of Research Results, Recruitment Lessons and Operational Considerations. *J Pers Med*. 2016;6(1). doi:10.3390/jpm6010002

© 2024 Wilcox H et al. *JAMA Network Open.*

17. McCarthy S, Das S, Kretzschmar W, et al. A reference panel of 64,976 haplotypes for genotype imputation. *Nat Genet*. 2016;48(10):1279-1283.

18. Loh PR, Danecek P, Palamara PF, et al. Reference-based phasing using the Haplotype Reference Consortium panel. *Nat Genet*. 2016;48(11):1443-1448.

19. Wang C, Zhan X, Liang L, Abecasis GR, Lin X. Improved ancestry estimation for both genotyping and sequencing data using projection procrustes analysis and genotype imputation. *Am J Hum Genet*. 2015;96(6):926-937.

20. Cann HM, de Toma C, Cazes L, et al. A human genome diversity cell line panel. *Science*. 2002;296(5566):261-262.

21. Wei WQ, Bastarache LA, Carroll RJ, et al. Evaluating phecodes, clinical classification software, and ICD-9-CM codes for phenome-wide association studies in the electronic health record. *PLoS One*. 2017;12(7):e0175508.

22. Dashti HS, Cade BE, Stutaite G, Saxena R, Redline S, Karlson EW. Sleep health, diseases, and pain syndromes: findings from an electronic health record biobank. *Sleep*. 2021;44(3). doi:10.1093/sleep/zsaa189

© 2024 Wilcox H et al. *JAMA Network Open.*
