# Supplementary material for: The Role of Circadian Rhythms and Sleep in Anorexia Nervosa
Source: JAMA Netw Open. 2024 Jan 4;7(1):e2350358. doi: 10.1001/jamanetworkopen.2023.50358 (PMC10767597; doi:10.1001/jamanetworkopen.2023.50358)
Supplement: Supplement 3. — Data Sharing Statement [file jamanetwopen-e2350358-s003.pdf]

Data Sharing Statement

Wilcox. The Role of Circadian Rhythms and Sleep in Anorexia Nervosa. *JAMA Netw Open*. Published January 04, 2024. doi:10.1001/jamanetworkopen.2023.50358

Data
Data available: No

Additional Information
Explanation for why data not available: Data included in the present study are publicly available as described in the manuscript.
